# Changing Empirical Antibiotic Prescribing Patterns Through a Clinical Decision Support System in Outpatient Paediatric Care in Italy: A Multicentre, Quality-Improvement Study

**DOI:** 10.3390/antibiotics15100989

**Published:** 2026-10-06

**Authors:** Giulia Brigadoi, Riccardo Boracchini, Cecilia Liberati, Lorenzo Chiusaroli, Marica De Pieri, Emelyne Gres, Lorenzo Tavernaro, Sofia Marchiorello, Luigi Cantarutti, Elisa Barbieri, Anna Cantarutti, Daniele Dona’, the CAPO-Project Group

**Affiliations:** 1 Pediatric Infectious Diseases Unit, Department of Women’s and Children’s Health, Padua University, 35128 Padua, Italy; 2 Division of Biostatistics, Epidemiology and Public Health, Department of Statistics and Quantitative Methods, University of Milan-Bicocca, 20126 Milan, Italy; 3 Department of Medicine and Surgery, University of Milan-Bicocca, 20900 Milan, Italy; 4 Pediatria e Neonatologia—UTIN, Azienda Ospedaliera Santa Maria di Terni, 05100 Terni, Italy; 5 Pediatric Infectious Diseases, Department of Women’s and Children’s Health, Azienda Ospedale Università Padova, 35128 Padova, Italy; 6 Infectious Diseases Unit, Santa Chiara Hospital, Azienda Sanitaria Universitaria Integrata del Trentino, 38122 Trento, Italy; 7 Società Servizi Telematici—Pedianet, 35128 Padova, Italy

**Keywords:** antimicrobial stewardship, clinical decision support systems, electronic health records, quality improvement, respiratory tract infections, paediatrics

## Abstract

Background/Objectives: Respiratory tract infections (RTIs) represent one of the most frequent reasons for antibiotic prescribing in paediatric primary care. Clinical decision support systems (CDSS) offer a strategy to promote guideline-concordant prescribing, but evidence from real-world implementation remains limited. Methods: We conducted a cluster-level controlled before-and-after quality-improvement study among 15 family care paediatricians of an Italian paediatric primary care network. Antibiotic prescriptions for RTIs in children aged < 14 years were compared between November 2023–April 2024 and November 2024–April 2025 across three groups: CDSS only, CDSS plus multilingual caregiver leaflets, and no intervention. The CDSS, integrated into the software where health records were recorded, provided guideline-based treatment pathways and weight-based dosing. Episodes were identified via ICD-9-CM codes and validated free text in the Pedianet database; log-binomial regression estimated relative risk with 95% CI. Within-paediatrician correlation was accounted for in the analysis using a cluster-robust variance estimator. Results: A total of 7138 RTI episodes were included. The availability of a CDSS integrated into the electronic health record system was associated with an increased use of guideline-recommended first-line antibiotics for RTIs, although the increase in the use of amoxicillin reached statistical significance only for CAP in both intervention groups. Non-prescription rates remained unchanged across the intervention groups. The control group also showed temporal changes, including increased non-prescription for pharyngitis and reduced amoxicillin use for CAP. Conclusions: CDSS integration was associated with increased first-line antibiotic use for certain conditions. However, its limited effect on overall prescribing volume underscores the persistent role of diagnostic uncertainty. Future stewardship strategies may benefit from combining CDSSs with point-of-care testing and behavioural interventions.

## 1. Introduction

In paediatric primary care, antibiotic prescribing for respiratory tract infections (RTIs) remains challenging. RTIs account for a substantial proportion of consultations and antibiotic prescriptions, although many episodes are viral, self-limiting, and unlikely to benefit from antibiotic treatment [1,2,3,4]. Prescribing decisions are often made with limited access to diagnostic testing or antimicrobial stewardship support, and follow-up may not always be readily available. In addition, parental expectations, time pressure, and concerns about potential complications may influence prescribing decisions and lead clinicians to prescribe antibiotics even when the indication is uncertain [5].

Inappropriate prescribing is not limited to unnecessary antibiotic use, but also includes the choice of broader-spectrum agents when narrower first-line options are recommended [5,6]. Antimicrobial stewardship programmes (ASPs) aim to improve both aspects of prescribing through evidence-based strategies. More broadly, ASPs in primary care encompass several dimensions, including appropriate antibiotic selection, weight-based dosing, and optimal treatment duration [7,8,9]. However, their implementation in the community differs from hospital-based implementation because primary care is more fragmented, prescribing practices vary considerably between clinicians, and opportunities for structured audits and feedback are more limited [10].

Several stewardship strategies have been evaluated in paediatric primary care settings, including clinical guidelines, audit and feedback, delayed prescribing, rapid diagnostic tests, clinician education, communication training, shared decision-making, and educational materials for families [11,12,13,14,15,16,17]. These interventions address different drivers of prescribing, from uncertainty in distinguishing viral from bacterial infections to caregiver expectations during consultations. Their effects are often modest when used in isolation, whereas multicomponent interventions, particularly those combining decision support with audit, feedback, or educational strategies, have generally shown more consistent benefits [18]. Maintaining these effects over time remains difficult, and the impact of outpatient stewardship interventions may decrease after implementation [19,20].

Real-world evidence on electronic stewardship interventions in Italian paediatric primary care remains limited. The present analysis was conducted within the CAPO project (Changing Antibiotic Prescribing in Outpatient paediatrics), a multicentre quality-improvement initiative promoted within the Pedianet primary-care network to evaluate strategies for improving empirical antibiotic prescribing for common paediatric infections. We therefore evaluated whether the availability of an electronic health record-integrated Clinical Decision Support System (CDSS), used alone or together with caregiver-directed educational materials, was associated with changes in empiric antibiotic prescribing for common paediatric RTIs.

## 2. Results

### 2.1. Study Population

A total of 7467 RTI episodes in 5126 children were included: 2052 episodes in Group A (CDSS only), 3238 in Group B (CDSS plus caregiver education with leaflets), and 2177 in Group C (control). Pharyngitis was the most frequent diagnosis (42.7%), followed by acute otitis media (AOM) (31.9%) and community-acquired pneumonia (CAP) (21.0%). Bronchiolitis and wheezing accounted for 329 episodes (4.4%) and were excluded due to the low number of prescriptions, leaving 7138 analysed episodes. Baseline demographic characteristics were broadly comparable across study groups and periods, with no statistically significant differences in sex distribution. Statistically significant differences in age, although clinically negligible, were observed in the pharyngitis subgroup across study groups, although median age remained approximately 7 years in all groups and did not differ between the pre- and post-implementation periods. An age difference between the pre- and post-implementation periods was also observed among children with CAP; however, the median age remained approximately 5 years across groups (Table 1 and Table S1–S5).

**Figure 1:**
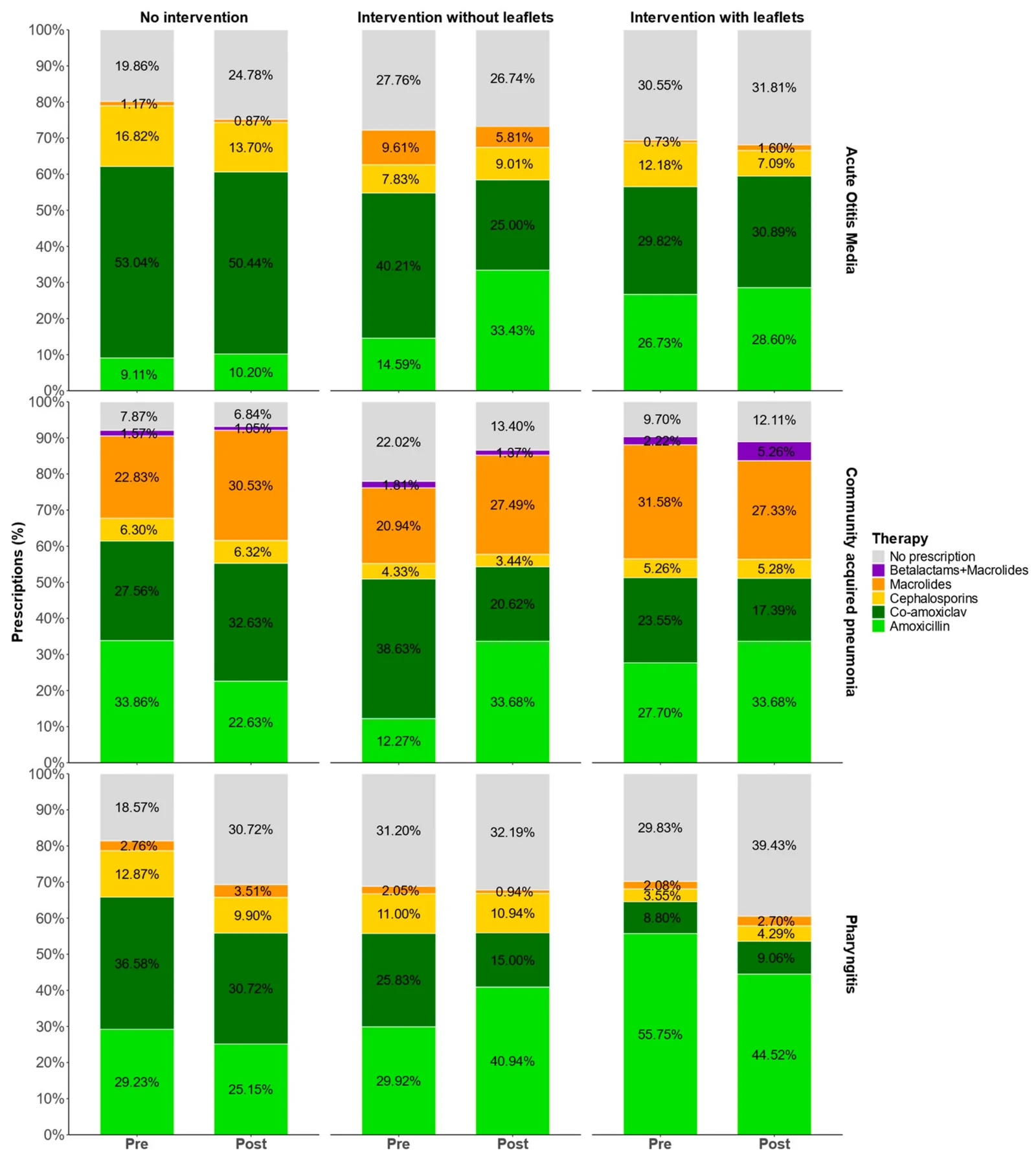
Distribution of antibiotic prescribing categories before and after the intervention, stratified by study group and diagnosis. Columns represent the control group (no intervention, Group C), the CDSS-only group (Group A), and the CDSS-plus-caregiver-leaflet group (Group B). Rows represent acute otitis media, community-acquired pneumonia, and pharyngitis/pharyngotonsillitis. Stacked bars show the proportion of episodes with no antibiotic prescription or with prescriptions for amoxicillin, co-amoxiclav, cephalosporins, macrolides, or a beta-lactam+macrolides combination during the pre-intervention period (November 2023–April 2024) and post-intervention period (November 2024–April 2025).

Overall prescribing patterns in the pre- and post-intervention periods are presented in Figure 1 and Table 2.

### 2.2. Pre–Post Comparison (Within-Group Comparisons)

#### 2.2.1. CDSS Only (Group A)

In the post-intervention period, Group A showed a shift toward guideline-recommended first-line antibiotics, most consistently for AOM and CAP. Amoxicillin prescribing increased for pharyngitis (from 29.9% to 40.9%; RR 1.24, 95% CI 0.69–2.24), AOM (from 14.6% to 33.4%; RR 2.29, 95% CI 1.04–5.06), and CAP (from 12.3% to 33.7%; RR 2.74, 95% CI 1.27–5.92); however, none remained significant after adjustment for the false discovery rate (FDR). These changes were accompanied by reductions in co-amoxiclav prescribing for pharyngitis (from 25.8% to 15.0%; RR 0.78, 95% CI 0.54–1.12), AOM (from 40.2% to 25.0%; RR 0.63, 95% CI 0.34–1.14), and CAP (from 38.6% to 20.6%; RR 0.47, 95% CI 0.28–0.78), reaching statistical significance only for CAP, which also remained significant after FDR adjustment. Non-prescription rates remained largely unchanged across conditions (Table 2, Figure 1).

#### 2.2.2. CDSS Plus Caregiver Leaflets (Group B)

Changes in the CDSS-plus-leaflets group were more modest and less consistent. For AOM, amoxicillin prescribing increased slightly from 26.7% to 28.6% (RR 1.11, 95% CI 1.02–1.20), with no significant change in co-amoxiclav use. For pharyngitis, amoxicillin prescribing decreased from 55.8% to 44.5% (RR 0.81, 95% CI 0.53–1.25), while non-prescription increased from 29.8% to 39.4% (RR 1.35, 95% CI 0.78–2.34). For CAP, amoxicillin prescribing increased from 27.7% to 33.2% (RR 1.32, 95% CI 1.08–1.60), a change that was statistically significant and remained so after FDR adjustment (Table 2, Figure 1).

#### 2.2.3. Control Group (Group C)

Temporal changes were also observed in the control group. For AOM, amoxicillin use remained low (9.1% vs. 10.2%; RR 1.13, 95% CI 0.50–2.54), and co-amoxiclav remained the predominant antibiotic (53.0% vs. 50.4%). For CAP, amoxicillin prescribing decreased from 33.3% to 22.6% (RR 0.68, 95% CI 0.37–1.24), although this change was not statistically significant. For pharyngitis, co-amoxiclav prescribing declined (from 36.6% to 30.7%; RR 0.72, 95% CI 0.68–0.76; significant after FDR adjustment) and non-prescription increased from 18.6% to 30.7% (RR 1.45, 95% CI 0.99–2.14).

### 2.3. Between-Group Comparisons

**Figure 2:**
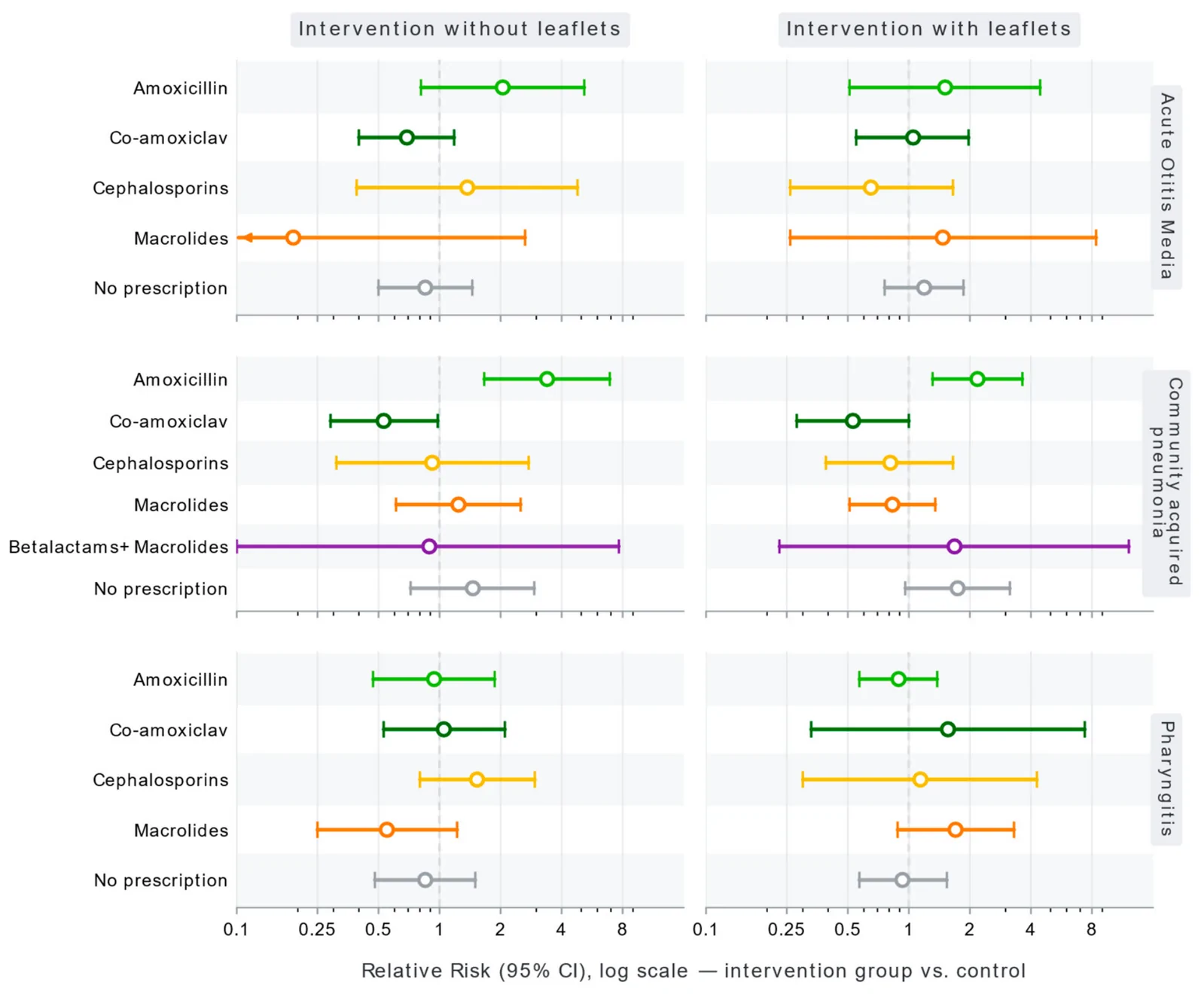
Risk ratio (RR) of receiving a specific antibiotic prescription for the intervention groups versus the control group, estimated from log-binomial models adjusted for the pre-intervention prescribing rate, with 95% CIs based on a paediatrician-clustered robust variance estimator.

Between-group comparisons are presented in Figure 2. Among children with AOM, amoxicillin prescribing tended to be higher in both intervention groups than in the control group (Group A: RR 2.05, 95% CI 0.81–5.17; Group B: RR 1.51, 95% CI 0.51–4.44). No antibiotic category differed significantly between the intervention groups and controls for AOM, including co-amoxiclav (Group A: RR 0.69, 95% CI 0.40–1.18; Group B: RR 1.05, 95% CI 0.55–1.97). No differences were observed in non-prescription rates (Group A: RR 0.85, 95% CI 0.50–1.45; Group B: RR 1.19, 95% CI 0.76–1.86).

Among children with CAP, amoxicillin prescribing was significantly higher in both intervention groups than in controls, with an approximately threefold increase in Group A (RR 3.39, 95% CI 1.66–6.91) and a twofold increase in Group B (RR 2.18, 95% CI 1.31–3.63); both associations remained significant after FDR adjustment. Co-amoxiclav prescribing was correspondingly lower in both groups (Group A: RR 0.53, 95% CI 0.29–0.98; Group B: RR 0.53, 95% CI 0.28–1.00), although these reductions did not remain significant after adjustment for the FDR.

Among children with pharyngitis, the distribution of prescribed antibiotics did not differ significantly between the intervention groups and the control group in any category.

A sensitivity analysis restricted to the first episode per child yielded results consistent with the main analysis for both the within-group and between-group comparisons (Supplementary Tables S6 and S7).

Forest plots show RRs with 95% CIs for each prescribing category in the CDSS-only group and in the CDSS-plus-caregiver-leaflet group, compared with the control group and adjusted for study period. Results are presented separately for acute otitis media, community-acquired pneumonia, and pharyngitis. *p*-values were adjusted for multiple comparisons using the Benjamini–Hochberg false discovery rate (FDR) within each diagnosis group; both unadjusted and FDR-adjusted *p*-values are reported in the Supplementary Materials Table S8. Because many comparisons were made, isolated results with borderline unadjusted *p*-values that do not survive FDR adjustment should be interpreted with caution.

## 3. Discussion

In this cluster-level controlled before-and-after quasi-experimental quality-improvement study conducted in Italian paediatric primary care, the availability of a CDSS integrated into the electronic health record system was associated with an increased use of guideline-recommended first-line antibiotics for RTIs, although the increase in the use of amoxicillin reached statistical significance only for CAP in both intervention groups. For CAP and AOM, narrow-spectrum antibiotics are generally recommended as first-line treatment when antibiotics are needed; nevertheless, broader-spectrum agents are still frequently prescribed because of concerns about treatment failure or the perceived need for broader coverage. In contrast, changes in overall antibiotic prescribing and non-prescription were less consistent across diagnoses and intervention groups. Overall, these findings suggest that, in routine primary care, a CDSS may have a greater influence on which antibiotic is prescribed than on the initial decision of whether to prescribe an antibiotic.

This distinction is relevant for antimicrobial stewardship. Although reducing unnecessary antibiotic use remains a key objective, inappropriate prescribing also includes the use of broader-spectrum agents when narrower alternatives are recommended. Broad-spectrum antibiotics are frequently prescribed in paediatric primary care despite the availability of narrower first-line options, as previously reported in both international and Italian studies [1,2]. Italian primary care data have also shown considerable variability in prescribing quality according to age, diagnosis, and clinical setting, highlighting the need for interventions that address not only the amount of antibiotic prescribing but also the choice of agent [21]. Our findings add to this evidence by showing that a CDSS embedded within the electronic health record may support antibiotic selection within the digital infrastructure used in routine clinical care.

The greater impact on antibiotic selection than on non-prescription use may reflect the practical challenges of decision-making in paediatric primary care. When there is uncertainty about the need for antibiotic treatment, a CDSS may be useful in guiding the choice of the recommended agent once the clinician has decided to prescribe, but it may be less able to resolve the initial diagnostic uncertainty. Integration within the prescribing workflow may be particularly useful in paediatrics, where weight-based dosing and age-specific recommendations add further complexity to treatment decisions. At the same time, the impact of digital decision support depends on factors such as usability, integration into routine workflow, clinician trust, uptake, and adaptation to the local setting [15,22,23,24]. These aspects could not be fully explored in our study, and neither diagnostic uncertainty nor clinicians’ reasons for prescribing were directly measured.

Findings in the CDSS-plus-leaflet group were more heterogeneous. The lack of an additional effect of caregiver educational materials might appear unexpected, as caregiver expectations and communication pressures are well-recognised influences on antibiotic prescribing [5,6]. However, prescribing patterns already differed between groups at baseline, with amoxicillin being prescribed more frequently in the intervention-plus-leaflet group during the pre-intervention period than in the other groups. In addition, the study did not record whether the educational materials were actually provided to caregivers, when they were delivered, or how they were used. The comparison therefore reflects the availability of the materials rather than a standardised caregiver-education intervention. The increase in non-prescription for pharyngitis remains of interest and may indicate that written information is particularly useful when viral aetiology is common and symptomatic management can be clearly explained. Previous studies suggest that written materials may be more effective when actively integrated into clinician–caregiver communication and shared decision-making [5,18,25,26,27]. However, non-prescription also increased in the control group; therefore, this finding should be considered exploratory. Similarly, the reduction in non-prescription in the intervention group without leaflets should be interpreted cautiously, particularly in light of the concurrent increase in non-prescription observed in the control group over the same period. Indeed, in the post-implementation period, non-prescription rates were approximately 30% in both groups, suggesting no substantial difference in absolute terms. These findings should also be considered in the context of the publication of the Italian national guidelines at the end of 2023 and their progressive dissemination and implementation throughout 2024, which may have influenced prescribing behaviour across all study groups independently of the intervention [28,29,30,31].

Different stewardship interventions address different reasons for inappropriate antibiotic use. Educational materials may help caregivers accept symptomatic management without antibiotics, audit and feedback may reinforce changes in clinicians’ prescribing behaviour, and diagnostic tools may reduce uncertainty at the point of care [10,26,32]. This last aspect is particularly important when a CDSS cannot resolve uncertainty because essential clinical or microbiological information is unavailable. Point-of-care tests, including C-reactive protein testing, rapid antigen detection tests for group A streptococcal pharyngitis, and rapid viral tests such as RSV antigen testing, may help guide treatment when used within evidence-based clinical pathways. Evidence from adult primary care and more recent paediatric studies suggests that point-of-care testing, alone or within broader stewardship interventions, may reduce antibiotic prescribing, although the magnitude of the effect varies across settings and depends on how the intervention is implemented [15,33,34,35]. Future interventions in Italian primary care could therefore assess whether integrating CDSS guidance with appropriately selected diagnostic tools produces larger reductions in unnecessary prescribing while preserving clinical safety.

The study has several strengths. First, it was conducted within a routine paediatric primary care network rather than under highly controlled trial conditions, making the findings relevant to real-world implementation. Second, the intervention was integrated into an existing electronic health record system used in routine practice, supporting its potential scalability. Third, the analysis focused on specific RTI diagnoses and antibiotic classes rather than relying only on aggregate antibiotic use, allowing changes in prescribing patterns to be interpreted more clinically. Finally, the inclusion of a contemporaneous control group provides useful context for interpreting changes observed in the intervention groups.

Several limitations should be acknowledged. First, the number of participating paediatricians was small, which may limit generalisability and make intervention-related changes difficult to separate from clinician-level prescribing habits, although the paediatricians were evenly distributed across northern, central, and southern Italy. Because each paediatrician contributed multiple episodes, observations were not fully independent; we accounted for within-paediatrician correlation using a cluster-robust variance estimator, and confidence intervals widened accordingly, but with only five clusters per arm, some residual imprecision remains, and estimates should be interpreted with caution. As paediatricians self-selected into the intervention arms, the groups differed at baseline, consistent with selection bias and confounding by indication, which the within-group pre–post design and the adjustment for the pre-intervention period mitigate but cannot fully remove.

Second, we evaluated prescribing patterns rather than per-episode guideline concordance, and clinical factors that may justify broader-spectrum treatment (allergy, recent antibiotic exposure, recurrence, vaccination status, disease severity) were not incorporated into the outcome. Relatedly, repeat episodes occurring more than 15 days after a previous episode could still represent persistence or recurrence rather than a new incident presentation. Nevertheless, a sensitivity analysis restricted to first episodes yielded consistent results. Diagnostic classification relied on EHR-recorded information, so some misclassification cannot be excluded.

Third, data on intervention uptake were lacking: we could not determine how often clinicians accessed the CDSS, followed its recommendations, used the dosing calculator, or shared the educational materials. We also did not assess prescribing dose accuracy, because dosing information in Pedianet is recorded only as non-standardised free text, precluding reliable comparisons either between the pre- and post-intervention periods or across study groups. Variation in intervention uptake may therefore have contributed to the observed between-group differences. Future studies should also examine how the intervention is used in everyday practice, including frequency of use, adherence to recommendations, and clinician satisfaction.

Finally, patient-level outcomes, including symptom duration, complications, emergency department visits, and hospital admissions, were not assessed. Information on emergency department visits and hospitalisations was not available for the entire study population, precluding reliable assessment of these outcomes across the full cohort. As a result, although the intervention was associated with a shift toward narrower-spectrum first-line agents for some conditions, we cannot determine whether this occurred without adverse effects. Future studies should therefore assess prescribing outcomes alongside relevant clinical and safety outcomes.

## 4. Materials and Methods

### 4.1. Study Population and Setting

The study was conducted within Pedianet [36,37], a nationwide network of Italian family paediatricians using the JuniorBit^®^ electronic health record system (JuniorBit® 7, So.Se.Pe Srl, Padua, Italy) in routine clinical practice, described in detail elsewhere [38]. The study population included children aged < 14 years presenting with an acute RTI under the care of the 15 paediatricians involved in the study, distributed across Northern (40%), Central (40%), and Southern (20%) Italy.

### 4.2. Study Design

This cluster-level controlled before-and-after quasi-experimental quality-improvement study evaluated the association between the availability of a CDSS, with or without caregiver-directed educational materials, and antibiotic prescribing patterns. The paediatrician represented the intervention cluster. Fifteen family paediatricians participated, with five included in each study group. Participation in the intervention groups was voluntary: five paediatricians received access to the CDSS alone (Group A), and five received access to the CDSS together with multilingual caregiver educational materials (Group B). Five additional paediatricians who continued usual care without access to either intervention served as the control group (Group C) and were not informed of the intervention. Paediatricians in the intervention groups attended a structured training session before implementation. Prescribing patterns were compared between the pre-intervention period, from 1 November 2023 to 30 April 2024, and the post-intervention period, from 1 November 2024 to 30 April 2025 and between groups.

### 4.3. CDSS Implementation

The CDSS, with or without caregiver-directed educational leaflets, became available in October 2024 and was integrated directly into the JuniorBit^®^ electronic medical record (Supplementary Materials Figure S1). It provided real-time, evidence-based clinical pathways for the diagnosis and management of pharyngitis, AOM, CAP, and bronchiolitis/wheezing. The pathways were based on national guidelines and designed to support clinicians in both diagnostic assessment and antibiotic selection. The tool also included an automated weight-based dosing calculator that generated antibiotic dose recommendations directly within the prescribing workflow.

In Group B, multilingual caregiver-directed educational materials were also made available in Italian, English, and French for the same clinical conditions. The materials provided practical guidance on home management, expected disease course, and indications for re-evaluation, with the aim of supporting appropriate healthcare use and acceptance of non-antibiotic management when indicated.

### 4.4. Case Definition and Outcome Assessment

RTI episodes were identified from JuniorBit^®^ records using ICD-9-CM diagnosis codes, the coding standard in use in Italy during the study period, together with information from free-text notes, the clinical diary, reasons for prescription, signs, and symptoms. Each episode was reviewed by three expert paediatricians (CL, LC, and MDP) and classified according to prespecified criteria as pharyngitis, AOM, CAP, or bronchiolitis/wheezing. Uncertain cases or disagreements regarding diagnostic classification were discussed with a fourth expert paediatrician (GB) and resolved by consensus.

Episodes in which antibiotics had already been prescribed by other healthcare providers, such as emergency departments or private paediatricians, as well as episodes with insufficient clinical information to allow classification according to the prespecified criteria, were excluded. A child could contribute more than one distinct RTI episode during the study period: assessments occurring within 15 days of an initial assessment were considered part of the same episode, whereas presentations more than 15 days apart were treated as separate episodes. The unit of analysis was therefore the RTI episode.

Antibiotic prescriptions were deduplicated at the patient-day level using Anatomical Therapeutic Chemical (ATC) codes and categorised as amoxicillin (J01CA04), co-amoxiclav (J01CR02), macrolides (J01FA*), and cephalosporins (J01D*).

The primary outcome was the prescribing pattern per RTI episode, defined as the proportion of episodes with no antibiotic prescription and the proportion receiving each antibiotic class. Changes were evaluated between the pre- and post-intervention periods within each study group and between the intervention groups and the control group.

### 4.5. Statistical Analysis

Descriptive statistics were used to summarise episode characteristics and prescribing patterns. Within each study group, changes in antibiotic prescribing between the pre- and post- intervention periods were assessed using Chi-square or Fisher’s exact tests for categorical variables. Log-binomial regression models were then applied to estimate relative risks (RRs) with 95% confidence intervals (95% CI) for non-prescription and for each antibiotic class, using the pre-intervention period as the reference. To evaluate differences in prescribing patterns between intervention groups, models were additionally fitted with group as the exposure, using the control group as the reference, adjusted for the pre-intervention prescribing rate. Results were presented overall and stratified by age class. Because episodes managed by the same paediatrician, including repeat episodes from the same child, cannot be regarded as fully independent, within-paediatrician correlation was accounted for in the analysis using a cluster-robust variance estimator. As a sensitivity analysis, models were refitted restricting the dataset to the first episode per child. Because multiple relative risks were estimated within each diagnosis (across antibiotic categories and study groups), we controlled the false discovery rate (FDR) using the Benjamini–Hochberg procedure. The correction was applied separately within each diagnostic group (AOM, CAP, pharyngitis), treating the full set of category-by-group relative-risk comparisons for that diagnosis as a single family of tests. Both unadjusted and FDR-adjusted *p*-values are reported, and a two-sided FDR-adjusted *p*-value < 0.05 was considered statistically significant. Given the descriptive, hypothesis-generating aim of the study, the adjusted *p*-values are intended to temper the interpretation of isolated comparisons rather than to define confirmatory findings.

Statistical significance was set at a level of 0.05. All statistical analyses were performed using SAS software, version 9.4 (SAS Institute, 17 Inc., Cary, NC, USA), and R Statistical Software version 4.5.1 (R Foundation for Statistical Computing, Vienna, Austria). Reporting followed the Standards for QUality Improvement Reporting Excellence (SQUIRE 2.0) guidelines [39].

### 4.6. Ethical Considerations

The study protocol and access to the Pedianet database were approved by the Pedianet Internal Scientific Committee. As the study was based on retrospectively collected anonymised data, no additional ethical approval or informed consent was required.

## 5. Conclusions

Embedding a CDSS within routine paediatric primary care was associated with a shift towards narrower-spectrum antibiotic use, with increased prescribing of amoxicillin, particularly for CAP. The CDSS seemed to have a greater effect on the choice of antibiotic than on the decision to prescribe antibiotics at all, suggesting that digital support may be particularly useful once treatment has been considered necessary. The additional contribution of caregiver-directed materials remains uncertain. Overall, these findings suggest that EHR-integrated CDSSs may be a useful component of paediatric outpatient antimicrobial stewardship, especially when supported by diagnostic tools and effective communication with caregivers.

## Figures and Tables

**Table 1:** Demographic characteristics of the population stratified by diagnosis.

	AOM			
	Group C	Group A	Group B	*p*-Value
	N = 771	N = 625	N = 987	
**Sex**				
**Female**	363 (47.08)	280 (44.8)	463 (46.91)	0.6407
**Male**	408 (52.92)	345 (55.2)	524 (53.09)	
**Age at diagnosis**	4.36 (2.56–6.69)	3.9 (2.18–6.23)	3.96 (2.43–6)	0.0458
**<2 years**	144 (18.68)	135 (21.6)	191 (19.35)	0.2216
**2–4 years**	318 (41.25)	264 (42.24)	454 (46)	
**5–6 years**	136 (17.64)	97 (15.52)	157 (15.91)	
**>=7 years**	173 (22.44)	129 (20.64)	185 (18.74)	
	**CAP**			
	**Group C**	**Group A**	**Group B**	***p*** **-value**
	**N = 317**	**N = 568**	**N = 683**	
**Sex**				
**Female**	152 (47.95)	272 (47.89)	335 (49.05)	0.9046
**Male**	165 (52.05)	296 (52.11)	348 (50.95)	
**Age at diagnosis**	4.84 (2.84–8.28)	4.55 (2.68–7.52)	4.51 (2.7–7.51)	0.2919
**<2 years**	46 (14.51)	96 (16.9)	106 (15.52)	0.6856
**2–4 years**	120 (37.85)	210 (36.97)	281 (41.14)	
**5–6years**	51 (16.09)	98 (17.25)	106 (15.52)	
**>=7 years**	100 (31.55)	164 (28.87)	190 (27.82)	
	**Pharyngitis**			
	**Group C**	**Group A**	**Group B**	***p*** **-value**
	**N = 1029**	**N = 711**	**N = 1447**	
**Sex**				
**Female**	475 (46.16)	322 (45.29)	721 (49.83)	0.0723
**Male**	554 (53.84)	389 (54.71)	726 (50.17)	
**Age at diagnosis**	6.64 (4.29–9.67)	6.7 (4.22–9.68)	7.44 (4.77–10.19)	<0.0001
**<2 years**	57 (5.54)	24 (3.38)	44 (3.04)	0.0005
**2–4 years**	281 (27.31)	208 (29.25)	349 (24.12)	
**5–6 years**	205 (19.92)	146 (20.53)	272 (18.8)	
**>=7 years**	486 (47.23)	333 (46.84)	782 (54.04)	

CAP, community-acquired pneumonia; AOM, acute otitis media; Group C, control group; Group A, CDSS only; Group B, CDSS plus caregiver education with leaflets.

**Table 2:** Relative risks with 95% cluster-robust CIs of antibiotic prescription within the different intervention groups, with the pre-intervention period as reference. *p*-values were adjusted for multiple comparisons using the Benjamini–Hochberg false discovery rate (FDR) within each diagnosis group; both unadjusted and FDR-adjusted *p*-values are shown. Because many comparisons were made, isolated results with borderline unadjusted *p*-values that do not survive FDR adjustment should be interpreted with caution.

Diagnosis Group	Therapy Strategy	All the Episodes								
		No Intervention			Intervention—No Leaflet			Intervention—Leaflet		
		RR (95%CI)	*p*-Value	ADJ	RR (95%CI)	*p*-Value	ADJ	RR (95%CI)	*p*-Value	ADJ
Acute Otitis Media	Amoxicillin	1.13 (0.5–2.54)	0.7759	0.8314	2.29 (1.04–5.06)	0.0397	0.119	1.11 (1.02–1.2)	0.0132	0.0655
	Co-amoxiclav	1.03 (0.89–1.19)	0.736	0.8314	0.63 (0.34–1.14)	0.1266	0.3166	0.99 (0.65–1.5)	0.954	0.954
	Cephalosporins	0.82 (0.48–1.39)	0.4624	0.7707	1.15 (1.02–1.29)	0.0175	0.0655	0.44 (0.34–0.58)	<0.001	<0.001
	Macrolides	0.76 (0.32–1.8)	0.5386	0.8078	0.67 (0.49–0.93)	0.0149	0.0655	1.72 (0.78–3.83)	0.1812	0.3398
	Betalactams + Macrolides	-	-	-	-	-	-	-	-	-
	No prescription	1.07 (0.77–1.48)	0.7007	0.8314	0.9 (0.53–1.54)	0.7022	0.8314	1.16 (0.95–1.42)	0.1561	0.3346
Community-acquired pneumonia	Amoxicillin	0.68 (0.37–1.24)	0.2075	0.415	2.74 (1.27–5.92)	0.0101	0.0608	1.32 (1.08–1.6)	0.0055	0.0491
	Co-amoxiclav	1.26 (0.6–2.67)	0.5433	0.6364	0.47 (0.28–0.78)	0.0036	0.0491	0.69 (0.5–0.94)	0.0189	0.0682
	Cephalosporins	0.92 (0.53–1.59)	0.7544	0.7544	0.68 (0.29–1.57)	0.3646	0.6081	0.68 (0.38–1.21)	0.1879	0.415
	Macrolides	1.23 (0.73–2.1)	0.4392	0.6081	1.44 (0.86–2.43)	0.1667	0.415	0.88 (0.57–1.36)	0.5657	0.6364
	Betalactams + Macrolides	0.67 (0.08–5.54)	0.7108	0.7526	0.78 (0.43–1.41)	0.4078	0.6081	1.9 (1.14–3.16)	0.0135	0.0609
	No prescription	0.81 (0.47–1.38)	0.4355	0.6081	0.72 (0.49–1.05)	0.0909	0.2728	1.25 (0.64–2.43)	0.5155	0.6364
Pharyngitis	Amoxicillin	1.09 (0.89–1.35)	0.403	0.6717	1.24 (0.69–2.24)	0.4771	0.7157	0.81 (0.53–1.25)	0.3439	0.6448
	Co-amoxiclav	0.72 (0.68–0.76)	<0.001	<0.001	0.78 (0.54–1.12)	0.1803	0.5408	0.86 (0.43–1.73)	0.6688	0.8361
	Cephalosporins	0.71 (0.38–1.33)	0.286	0.6129	1 (0.6–1.69)	0.9932	0.9932	0.97 (0.61–1.57)	0.9133	0.9932
	Macrolides	1.18 (0.7–1.98)	0.5367	0.7318	0.56 (0.24–1.27)	0.1655	0.5408	1.48 (0.87–2.51)	0.1475	0.5408
	Betalactams + Macrolides	-	-	-	-	-	-	-	-	-
	No prescription	1.45 (0.99–2.14)	0.0575	0.4316	1 (0.57–1.78)	0.9924	0.9932	1.35 (0.78–2.34)	0.2805	0.6129

## Data Availability

The data used in this study cannot be made available in a public repository due to Italian data protection laws. The anonymised datasets generated and/or analysed during the current study can be provided upon reasonable request from the corresponding author after written approval by the Internal Scientific Committee (info@pedianet.it).

## Supplementary Materials

The following supporting information can be downloaded at https://www.mdpi.com/article/10.3390/antibiotics15100989/s1: Figure S1. Implementation and clinical workflow of the CAPO clinical decision support system within the JuniorBit^®^ electronic health record, Table S1: Demographic characteristics of the overall population in the three groups; Table S2: Demographic characteristics of the overall population in the three groups and by periods; Table S3: Demographic characteristics of the population included in the analysis for AOM in the three groups and by periods; Table S4: Demographic characteristics of the population included in the analysis for CAP in the three groups and by periods; Table S5: Demographic characteristics of the population included in the analysis for pharyngitis in the three groups and by periods; Table S6. Relative risks with 95% cluster-robust CIs of antibiotic prescription within the different intervention groups with the pre-intervention period as reference for the first episode only; Table S7. Relative risks of receiving a specific antibiotic prescription for the intervention groups versus the control group, estimated from log-binomial models adjusted for the pre-intervention prescribing rate, with 95% CIs based on a paediatrician-clustered robust variance estimator; Table S8. Relative risks of receiving a specific antibiotic prescription for the intervention groups versus the control group, estimated from log-binomial models adjusted for the pre-intervention prescribing rate, with 95% CIs based on a paediatrician-clustered robust variance estimator for the first episode only.

## Abbreviations

The following abbreviations are used in this manuscript:

ASP	Antimicrobial Stewardship Programme
CAP	Community-acquired Pneumonia
AOM	Acute Otitis Media
RR	Relative Risk
CI	Confidence Interval
RTIs	Respiratory Tract Infections
CDSS	Clinical Decision Support System
