# Supplementary material for: Changing Empirical Antibiotic Prescribing Patterns Through a Clinical Decision Support System in Outpatient Paediatric Care in Italy: A Multicentre, Quality-Improvement Study
Source: Antibiotics (Basel). 2026 Oct 6;15(10):989. doi: 10.3390/antibiotics15100989 (PMC13700756; doi:10.3390/antibiotics15100989)
Supplement: Supplementary file 1 [file antibiotics-15-00989-s001.zip › antibiotics-4533118-supplementary.pdf]

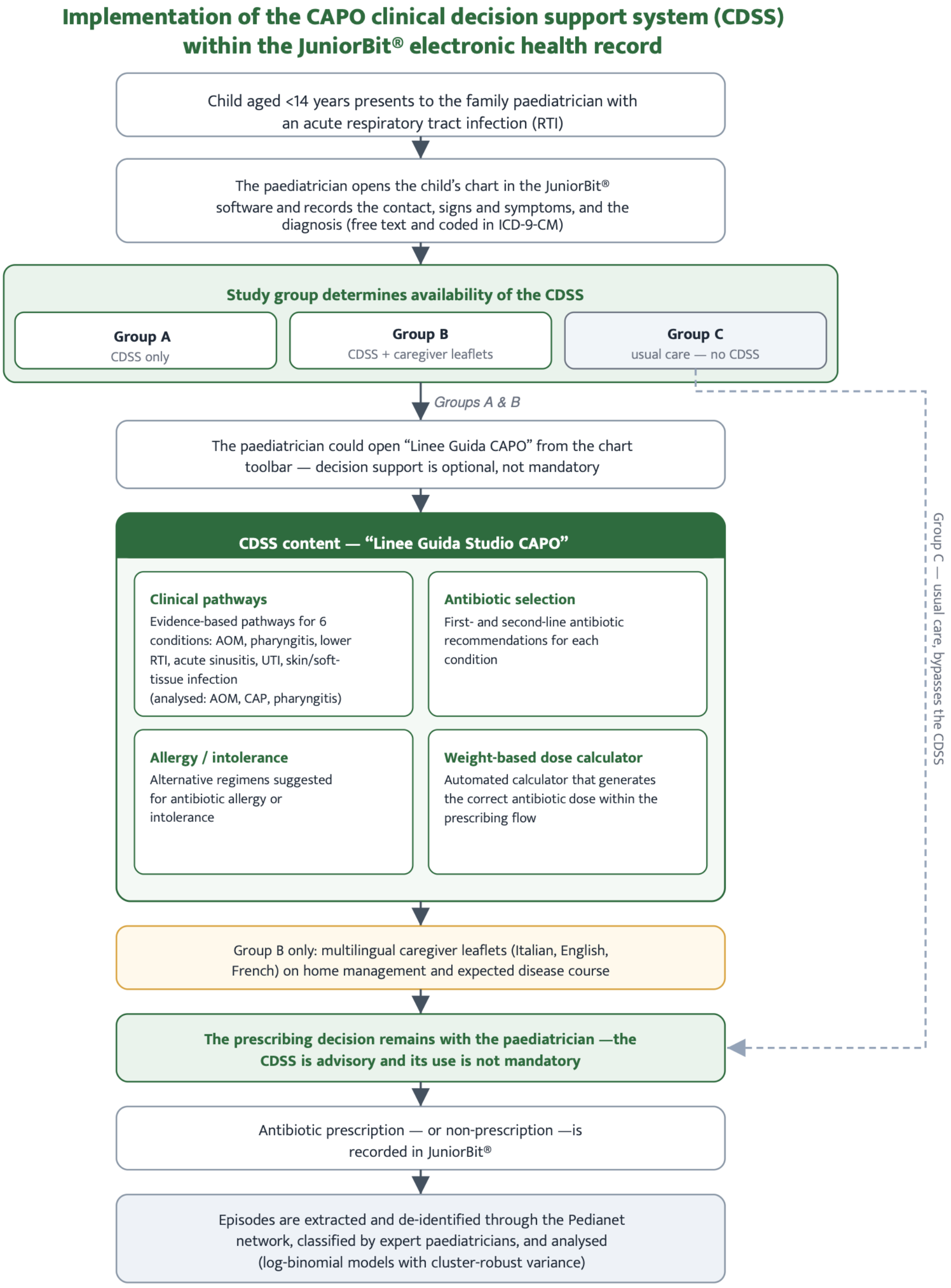

Figure S1. Implementation and clinical workflow of the CAPO clinical decision support system within the JuniorBit® electronic health record

Table S1. Demographic characteristics of the overall population in the three groups

	Overall			
	Group C<br>N = 2177	Group A<br>N = 2052	Group B<br>N = 3238	P-value
**Sex**				
**Female**	1018 (46.76)	933 (45.47)	1569 (48.46)	0.0964
**Male**	1159 (53.24)	1119 (54.53)	1669 (51.54)	
**Diagnosis**				
**Bronchiolitis / Wheezing**	60 (2.76)	148 (7.21)	121 (3.74)	<0.0001
**Acute otitis media**	771 (35.42)	625 (30.46)	987 (30.48)	
**Community acquired pneumonia**	317 (14.56)	568 (27.68)	683 (21.09)	
**Pharyngitis**	1029 (47.27)	711 (34.65)	1447 (44.69)	
**Age at diagnosis**	5.37 (3.24 - 8.53)	4.85 (2.84 - 7.86)	5.32 (3.18 - 8.71)	<0.0001
**< 2 years**	279 (12.82)	328 (15.98)	410 (12.66)	0.0004
**2 - < 5 years**	729 (33.49)	730 (35.58)	1111 (34.31)	
**5 - < 7 years**	398 (18.28)	354 (17.25)	544 (16.8)	
**>= 7 years**	771 (35.42)	640 (31.19)	1173 (36.23)	

Table S2. Demographic characteristics of the overall population in the three groups and by periods

	No intervention			Intervention			Intervention leaflet		
	Pre N = 1119	Post N = 1058	P-value	Pre N = 1008	Post N = 1044	P-value	Pre N = 1796	Post N = 1442	P-value
**Sex**									
**Female**	535 (47.81)	483 (45.65)	0.3131	459 (45.54)	474 (45.4)	0.9516	857 (47.72)	712 (49.38)	0.3479
**Male**	584 (52.19)	575 (54.35)		549 (54.46)	570 (54.6)		939 (52.28)	730 (50.62)	
**Diagnosis**									
**Bronchiolitis / Wheezing**	20 (1.79)	40 (3.78)	<.0001	59 (5.85)	89 (8.52)	0.0002	67 (3.73)	54 (3.74)	0.4617
**Acute otitis media**	428 (38.25)	343 (32.42)		281 (27.88)	344 (32.95)		550 (30.62)	437 (30.31)	
**Community acquired pneumonia**	127 (11.35)	190 (17.96)		277 (27.48)	291 (27.87)		361 (20.10)	322 (22.33)	
**Pharyngitis**	544 (48.61)	485 (45.84)		391 (38.79)	320 (30.65)		818 (45.55)	629 (43.62)	
**Age at diagnosis**	5.29 (3.28 - 8.50)	5.45 (3.17 - 8.60)	0.4286	4.85 (2.78 - 7.72)	4.85 (2.93 - 7.91)	0.3566	5.33 (3.17 - 8.75)	5.31 (3.20 - 8.68)	0.847
**< 2 years**	141 (12.6)	138 (13.04)	0.4366	165 (16.37)	163 (15.61)	0.5964	246 (13.7)	164 (11.37)	0.0734
**2 - < 5 years**	391 (34.94)	338 (31.95)		359 (35.62)	371 (35.54)		588 (32.74)	523 (36.27)	
**5 - < 7 years**	194 (17.34)	204 (19.28)		182 (18.06)	172 (16.48)		311 (17.32)	233 (16.16)	
**>= 7 years**	393 (35.12)	378 (35.73)		302 (29.96)	338 (32.38)		651 (36.25)	522 (36.2)	

Table S3. Demographic characteristics of the population included in the analysis for AOM in the three groups and by periods

	No intervention			Intervention			Intervention leaflet		
	Pre N = 428	Post N = 343	P-value	Pre N = 281	Post N = 344	P-value	Pre N = 550	Post N = 437	P-value
**Sex**									
**Female**	214 (50)	149 (43.44)	0.0698	127 (45.2)	153 (44.48)	0.8573	241 (43.82)	222 (50.8)	0.029
**Male**	214 (50)	194 (56.56)		154 (54.8)	191 (55.52)		309 (56.18)	215 (49.2)	
**Age at diagnosis**	4.21 (2.59 - 6.6)	4.48 (2.49 - 6.9)	0.6366	3.75 (2.11 - 5.84)	3.98 (2.25 - 6.52)	0.3004	4.02 (2.48 - 5.74)	3.85 (2.41 - 6.29)	0.8776
**< 2 years**	78 (18.22)	66 (19.24)	0.7376	61 (21.71)	74 (21.51)	0.3918	109 (19.82)	82 (18.76)	0.083
**2 - < 5 years**	184 (42.99)	134 (39.07)		128 (45.55)	136 (39.53)		244 (44.36)	210 (48.05)	
**5 - < 7 years**	74 (17.29)	62 (18.08)		40 (14.23)	57 (16.57)		101 (18.36)	56 (12.81)	
**>= 7 years**	92 (21.5)	81 (23.62)		52 (18.51)	77 (22.38)		96 (17.45)	89 (20.37)	

Table S4. Demographic characteristics of the population included in the analysis for CAP in the three groups and by periods

	No intervention			Intervention			Intervention leaflet		
	Pre N = 127	Post N = 190	P-value	Pre N = 227	Post N = 291	P-value	Pre N = 361	Post N = 322	P-value
**Sex**									
**Female**	58 (45.67)	94 (49.47)	0.5064	140 (50.54)	132 (45.36)	0.2167	183 (50.69)	152 (47.2)	0.3628
**Male**	69 (54.33)	96 (50.53)		137 (49.46)	159 (54.64)		178 (49.31)	170 (52.8)	
**Age at diagnosis**	3.97 (2.47 - 6.42)	5.53 (3.11 - 9.54)	0.004	4.15 (2.45 - 6.57)	5.46 (3.04 - 8.49)	0.003	4.4 (2.51 - 7)	4.67 (2.93 - 8.21)	0.0384
**< 2 years**	23 (18.11)	23 (12.11)	0.0045	52 (18.77)	44 (15.12)	0.0005	68 (18.84)	38 (11.8)	0.0513
**2 - < 5 years**	59 (46.46)	61 (32.11)		120 (43.32)	90 (30.93)		147 (40.72)	134 (41.61)	
**5 - < 7 years**	17 (13.39)	34 (17.89)		46 (16.61)	52 (17.87)		56 (15.51)	50 (15.53)	
**>= 7 years**	28 (22.05)	72 (37.89)		59 (21.3)	105 (36.08)		90 (24.93)	100 (31.06)	

Table S5. Demographic characteristics of the population included in the analysis for pharyngitis in the three groups and by periods

	No intervention			Intervention			Intervention leaflet		
	**Pre N = 544**	**Post N = 485**	**P-value**	**Pre N = 391**	**Post N = 320**	**P-value**	**Pre N = 818**	**Post N = 629**	**P-value**
**Sex**									
**Female**	249 (45.77)	226 (46.6)	0.7908	174 (44.5)	148 (46.25)	0.6412	404 (49.39)	317 (50.4)	0.7036
**Male**	295 (54.23)	259 (53.4)		217 (55.5)	172 (53.75)		414 (50.61)	312 (49.6)	
**Age at diagnosis**	6.91 (4.37 - 9.61)	6.16 (4.18 - 9.83)	0.3388	6.78 (4.37 - 9.7)	6.62 (4.04 - 9.65)	0.6394	7.54 (4.87 - 10.18)	7.16 (4.63 - 10.22)	0.2683
**< 2 years**	25 (4.6)	32 (6.6)	0.2512	15 (3.84)	9 (2.81)	0.153	24 (2.93)	20 (3.18)	0.4441
**2 - < 5 years**	147 (27.02)	134 (27.63)		102 (26.09)	106 (33.13)		187 (22.86)	162 (25.76)	
**5 - < 7 years**	102 (18.75)	103 (21.24)		88 (22.51)	58 (18.13)		150 (18.34)	122 (19.4)	
**>= 7 years**	270 (49.63)	216 (44.54)		186 (47.57)	147 (45.94)		457 (55.87)	325 (51.67)	

Table S6. Relative Risks with 95% cluster-robust CIs of antibiotic prescription within the different intervention groups with the pre-intervention period as reference for the first episode only.

P-values were adjusted for multiple comparisons using the Benjamini–Hochberg false discovery rate (FDR) within each diagnosis group; both unadjusted and FDR-adjusted p-values are shown. Because many comparisons were made, isolated results with borderline unadjusted p-values that do not survive FDR adjustment should be interpreted with caution.

Diagnosis group	Therapy strategy	First episode for children								
		No Intervention			Intervention - No leaflet			Intervention - Leaflet		
		RR (95%CI)	P-value	ADJ	RR (95%CI)	P-value	ADJ	RR (95%CI)	P-value	ADJ
AOM	Amoxicillin	1.20 (0.5 - 2.93)	0,6811	0,796142308	2.29 (1.07 - 4.91)	0,03238	0,121425	1.15 (1.04 - 1.27)	0,00531	0,039825
	Co-amoxiclav	1.04 (0.94 - 1.15)	0,44405	0,666075	0.65 (0.37 - 1.14)	0,13198	0,2474625	0.97 (0.61 - 1.56)	0,91235	0,91235
	Cephalosporins	0.71 (0.47 - 1.08)	0,10686	0,228985714	1.04 (0.99 - 1.08)	0,10413	0,228985714	0.42 (0.29 - 0.59)	< 0.001	< 0.001
	Macrolides	0.89 (0.38 - 2.08)	0,79391	0,850617857	0.7 (0.52 - 0.95)	0,02408	0,1204	1.94 (0.88 - 4.32)	0,10241	0,228985714
	Betalactams + Macrolides	-	-	-	-	-	-	-	-	-
	No prescription	1.09 (0.8 - 1.5)	0,57608	0,785563636	0.89 (0.51 - 1.55)	0,68999	0,796142308	1.15 (0.89 - 1.47)	0,28065	0,46775
CAP	Amoxicillin	0.65 (0.38 - 1.08)	0,098575	0,253478571	2.86 (1.27 - 6.43)	0,011371	0,042912	1.32 (1.12 - 1.56)	0,000913	0,016434
	Co-amoxiclav	1.3 (0.61 - 2.76)	0,49431	0,635541429	0.47 (0.27 - 0.82)	0,00771	0,042912	0.67 (0.5 - 0.92)	0,01192	0,042912
	Cephalosporins	0.94 (0.55 - 1.6)	0,82551	0,68097375	0.56 (0.24 - 1.3)	0,17653	0,3971925	0.7 (0.38 - 1.29)	0,2528	0,420725455
	Macrolides	1.24 (0.69 - 2.22)	0,47355	0,635541429	1.4 (0.81 - 2.44)	0,23233	0,420725455	0.89 (0.59 - 1.33)	0,5592	0,67104
	Betalactams + Macrolides	0.7 (0.08 - 5.94)	0,74151	0,68097375	0.63 (0.29 - 1.4)	0,25711	0,420725455	2.07 (1.3 - 3.28)	0,00204	0,01836
	No prescription	0.74 (0.41 - 1.32)	0,30223	0,453345	0.73 (0.51 - 1.05)	0,09321	0,253478571	1.19 (0.61 - 2.32)	0,60531	0,68097375
Pharyngitis	Amoxicillin	1.08 (0.87 - 1.34)	0,48457	0,726855	1.26 (0.68 - 2.35)	0,46836	0,726855	0.83 (0.54 - 1.26)	0,38395	0,71990625
	Co-amoxiclav	0.73 (0.67 - 0.8)	<0.001	<0.001	0.78 (0.53 - 1.16)	0,22265	0,556625	0.87 (0.43 - 1.76)	0,69609	0,803180769
	Cephalosporins	0.65 (0.4 - 1.04)	0,06987	0,3247875	1.05 (0.67 - 1.65)	0,83472	0,894342857	0.89 (0.54 - 1.48)	0,663	0,803180769
	Macrolides	1.15 (0.7 - 1.88)	0,57743	0,787404545	0.42 (0.11 - 1.56)	0,19507	0,556625	1.61 (0.93 - 2.76)	0,08661	0,3247875
	Betalactams + Macrolides	-	-	-	-	-	-	-	-	-
	No prescription	1.47 (1.02 - 2.13)	0,03903	0,292725	0.97 (0.53 - 1.78)	0,91963	0,91963	1.32 (0.75 - 2.3)	0,3359	0,719785714

Table S7. Relative Risks of receiving a specific antibiotic prescription for the intervention groups versus the control group, estimated from log-binomial models adjusted for the pre-intervention prescribing rate, with 95% CIs based on a paediatrician-clustered robust variance estimator.
P-values were adjusted for multiple comparisons using the Benjamini–Hochberg false discovery rate (FDR) within each diagnosis group; both unadjusted and FDR-adjusted p-values are shown. Because many comparisons were made, isolated results with borderline unadjusted p-values that do not survive FDR adjustment should be interpreted with caution.

Diagnosis group	Therapy strategy	First episode for children					
		Intervention - No leaflet			Intervention - Leaflet		
		RR (95%CI)	Unadjusted P-value	FDR-adjusted P-value	RR (95%CI)	Unadjusted P-value	FDR-adjusted P-value
AOM	Amoxicillin	1.97 (0.78 - 4.97)	0,14926	0,7033	1.51 (0.53 - 4.35)	0,44224	0,715725
	Co-amoxiclav	0.69 (0.41 - 1.18)	0,17443	0,7033	1.02 (0.52 - 1.99)	0,95548	0,95548
	Cephalosporins	1.67 (0.54 - 5.2)	0,37684	0,715725	0.79 (0.35 - 1.78)	0,57258	0,715725
	Macrolides	0.18 (0.01 - 2.61)	0,21099	0,7033	1.38 (0.25 - 7.79)	0,71396	0,793288889
	Betalactams + Macrolides	-	-	-	-	-	-
	No prescription	0.85 (0.5 - 1.45)	0,55988	0,715725	1.18 (0.75 - 1.88)	0,47128	0,715725
CAP	Amoxicillin	3.49 (1.84 - 6.59)	0,000120168	0,001442016	2.22 (1.44 - 3.44)	0,000333347	0,002000082
	Co-amoxiclav	0.53 (0.29 - 0.98)	0,041996	0,1007904	0.5 (0.27 - 0.94)	0,030765	0,092295
	Cephalosporins	0.69 (0.25 - 1.87)	0,46449	0,69208	0.79 (0.39 - 1.63)	0,52864	0,69208
	Macrolides	1.19 (0.56 - 2.55)	0,64673	0,69208	0.83 (0.51 - 1.38)	0,47791	0,69208
	Betalactams + Macrolides	0.64 (0.07 - 5.87)	0,69208	0,69208	1.74 (0.24 - 12.5)	0,58173	0,69208
	No prescription	1.72 (0.98 - 3)	0,057741	0,115482	1.88 (1.07 - 3.31)	0,028937	0,092295
Pharyngitis	Amoxicillin	0.95 (0.46 - 1.97)	0,88519	0,88802	0.92 (0.59 - 1.43)	0,70396	0,88802
	Co-amoxiclav	-	-	-	-	-	-
	Cephalosporins	1.63 (0.96 - 2.78)	0,07128	0,28512	1.09 (0.31 - 3.82)	0,88802	0,88802
	Macrolides	0.43 (0.12 - 1.51)	0,18831	0,50216	1.87 (0.95 - 3.67)	0,06834	0,28512
	Betalactams + Macrolides	-	-	-	-	-	-
	No prescription	0.8 (0.44 - 1.47)	0,47151	0,88802	0.87 (0.52 - 1.45)	0,58953	0,88802

Table S8. Relative Risks of receiving a specific antibiotic prescription for the intervention groups versus the control group, estimated from log-binomial models adjusted for the pre-intervention prescribing rate, with 95% CIs based on a paediatrician-clustered robust variance estimator for the first episode only.

P-values were adjusted for multiple comparisons using the Benjamini–Hochberg false discovery rate (FDR) within each diagnosis group; both unadjusted and FDR-adjusted p-values are shown. Because many comparisons were made, isolated results with borderline unadjusted p-values that do not survive FDR adjustment should be interpreted with caution.

Diagnosis group	Therapy strategy	All the episodes					
		Intervention - No leaflet			Intervention - Leaflet		
		RR (95%CI)	Unadjusted P-value	FDR-adjusted P-value	RR (95%CI)	Unadjusted P-value	FDR-adjusted P-value
AOM	Amoxicillin	2.05 (0.81 - 5.17)	0,12897	0,64644	1.51 (0.51 - 4.44)	0,4523	0,64644
	Co-amoxiclav	0.69 (0.4 - 1.18)	0,17312	0,64644	1.05 (0.55 - 1.97)	0,89124	0,724298182
	Cephalosporins	1.37 (0.39 - 4.79)	0,61913	0,724298182	0.65 (0.26 - 1.65)	0,36229	0,64644
	Macrolides	0.19 (0.01 - 2.64)	0,21548	0,64644	1.47 (0.26 - 8.38)	0,66394	0,724298182
	Betalactams + Macrolides	-	-	-	-	-	-
	No prescription	0.85 (0.5 - 1.45)	0,5583	0,724298182	1.19 (0.76 - 1.86)	0,4529	0,724298182
CAP	Amoxicillin	3.39 (1.66 - 6.91)	0,000788429	0,009461148	2.18 (1.31 - 3.63)	0,00269607	0,01617642
	Co-amoxiclav	0.53 (0.29 - 0.98)	0,042089	0,150849	0.53 (0.28 - 1)	0,050283	0,150849
	Cephalosporins	0.92 (0.31 - 2.75)	0,88579	0,91416	0.81 (0.39 - 1.65)	0,55481	0,726252
	Macrolides	1.24 (0.61 - 2.51)	0,54755	0,726252	0.83 (0.51 - 1.35)	0,4554	0,726252
	Betalactams + Macrolides	0.89 (0.1 - 7.66)	0,91416	0,91416	1.68 (0.23 - 12.16)	0,60521	0,726252
	No prescription	1.46 (0.72 - 2.93)	0,29052	0,58104	1.74 (0.96 - 3.15)	0,06892	0,165408
Pharyngitis	Amoxicillin	0.94 (0.47 - 1.87)	0,85425	0,87903	0.89 (0.57 - 1.38)	0,59458	0,87903
	Co-amoxiclav	1.05 (0.53 - 2.1)	0,87903	0,87903	1.56 (0.33 - 7.37)	0,5711	0,87903
	Cephalosporins	1.53 (0.8 - 2.95)	0,20085	0,48204	1.14 (0.3 - 4.28)	0,85147	0,87903
	Macrolides	0.55 (0.25 - 1.22)	0,14313	0,42939	1.7 (0.88 - 3.3)	0,11678	0,42939
	Betalactams + Macrolides	-	-	-	-	-	-
	No prescription	0.85 (0.48 - 1.5)	0,58359	0,87903	0.93 (0.57 - 1.54)	0,79011	0,87903

Revised Standards for Quality Improvement Reporting Excellence (SQUIRE 2.0) Checklist

Text Section and Item Name	Section or Item Description	Page
**Title and Abstract**		
**1. Title**	Indicate that the manuscript concerns an initiative to improve healthcare (broadly defined to include the quality, safety, effectiveness, patient-centeredness, timeliness, cost, efficiency, and equity of healthcare)	1
**2. Abstract**	a. Provide adequate information to aid in searching and indexing<br>b. Summarize all key information from various sections of the text using the abstract format of the intended publication or a structured summary such as: background, local problem, methods, interventions, results, conclusions	1-2
**Introduction**	*Why did you start?*	
3. Problem Description	Nature and significance of the local problem	2
4. Available knowledge	Summary of what is currently known about the problem, including relevant previous studies	2
5. Rationale	Informal or formal frameworks, models, concepts, and/or theories used to explain the problem, any reasons or assumptions that were used to develop the intervention(s), and reasons why the intervention(s) was expected to work	2
6. Specific aims	Purpose of the project and of this report	2
**Methods**	What did you do?	
7. Context	Contextual elements considered important at the outset of introducing the intervention(s)	13
8. Intervention(s)	a. Description of the intervention(s) in sufficient detail that others could reproduce it<br>b. Specifics of the team involved in the work	13
9. Study of the Intervention(s)	a. Approach chosen for assessing the impact of the intervention(s)<br>b. Approach used to establish whether the observed outcomes were due to the intervention(s)	13-14
10. Measures	a. Measures chosen for studying processes and outcomes of the intervention(s), including rationale for choosing them, their operational definitions, and their validity and reliability<br>b. Description of the approach to the ongoing assessment of contextual elements that contributed to the success, failure, efficiency, and cost<br>c. Methods employed for assessing completeness and accuracy of data	13-14
11. Analysis	a. Qualitative and quantitative methods used to draw inferences from the data<br>b. Methods for understanding variation within the data, including the effects of time as a variable	14

12. Ethical Considerations	Ethical aspects of implementing and studying the intervention(s) and how they were addressed, including, but not limited to, formal ethics review and potential conflict(s) of interest	14
**Results**	What did you find?	
13. Results	a. Initial steps of the intervention(s) and their evolution over time (e.g., time-line diagram, flow chart, or table), including modifications made to the intervention during the project<br>b. Details of the process measures and outcome<br>c. Contextual elements that interacted with the intervention(s)<br>d. Observed associations between outcomes, interventions, and relevant contextual elements<br>e. Unintended consequences such as unexpected benefits, problems, failures, or costs associated with the intervention(s).<br>f. Details about missing data	3-9
**Discussion**	What does it mean?	
14. Summary	a. Key findings, including relevance to the rationale and specific aims<br>b. Particular strengths of the project	10-12
15. Interpretation	a. Nature of the association between the intervention(s) and the outcomes<br>b. Comparison of results with findings from other publications<br>c. Impact of the project on people and systems<br>d. Reasons for any differences between observed and anticipated outcomes, including the influence of context<br>e. Costs and strategic trade-offs, including opportunity costs	10-12
16. Limitations	a. Limits to the generalizability of the work<br>b. Factors that might have limited internal validity such as confounding, bias, or imprecision in the design, methods, measurement, or analysis<br>c. Efforts made to minimize and adjust for limitations	11-12
17. Conclusions	a. Usefulness of the work<br>b. Sustainability<br>c. Potential for spread to other contexts<br>d. Implications for practice and for further study in the field<br>e. Suggested next steps	15
**Other information**		
18. Funding	Sources of funding that supported this work. Role, if any, of the funding organization in the design, implementation, interpretation, and reporting	15
